# Lupus Miliaris Disseminatus Faciei: A Report of a Rare Case and Its Differential Diagnosis

**DOI:** 10.7759/cureus.63245

**Published:** 2024-06-26

**Authors:** Tarang Patel, Yashdeep Singh Pathania, Parth R Goswami, Gyanendra Singh, Rushang Dave

**Affiliations:** 1 Pathology, All India Institute of Medical Sciences (AIIMS) Rajkot, Rajkot, IND; 2 Dermatology, Venereology and Leprology, All India Institute of Medical Sciences (AIIMS) Rajkot, Rajkot, IND; 3 Pathology, Shantabaa Medical College and General Hospital Amreli, Amreli, IND

**Keywords:** cutaneous sarcoidosis, granulomatous rosacea, periorbital papules, cutaneous granuloma, lupus miliaris disseminatus faciei

## Abstract

Lupus miliaris disseminatus faciei (LMDF), often known as "acne agminata," is an uncommon illness that causes facial papules. Clinically, it has monomorphic reddish-brown, dome-shaped central papules with periorbital location. Histopathologically, a cutaneous granulomatous response is common around hair follicles and is accompanied by central necrosis. In the dermatology outpatient clinic, a 51-year-old woman had many tiny papules on her right side malar area for one to two months. Few of them started to regress and demonstrated healing with superficial scarring. The pathology showed a granulomatous response on microscopy, and histology and clinical correlation confirmed the case as Lupus miliaris disseminatus faciei. LMDF must be distinguished from tuberculous granuloma, granulomatous rosacea, and perioral dermatitis. The patient was prescribed systemic dapsone and topical tacrolimus therapy, and the lesion improved at the follow-up visit.

## Introduction

Lupus miliaris disseminatus faciei (LMDF) is a skin disorder characterized by the presence of granulomas on the face. This atypical skin condition is often challenging to diagnose and manage. It bears a striking resemblance to other granulomatous disorders, such as sarcoidosis and rosacea. The distinctive clinical presentation and histological characteristics of LMDF differentiate it from other illnesses, despite the unknown origin of the disease [1]. In 1878, Fox first documented LMDF [2]. There have been around 200 cases documented in the literature [3].

Various literature suggests that this condition is equivalent to granulomatous rosacea [4,5]. The disorder has recently been named facial idiopathic granulomata with regressive evolution. The condition typically presents as uniform yellowish-brown papules, primarily affecting the centre of the face, with a tendency to occur around the eyes. Occasionally, the sickness only impacts the canthus and eyelids. Observing involvement of the upper limb or axillae is a rare occurrence [4-6].

## Case presentation

**Figure 1:**
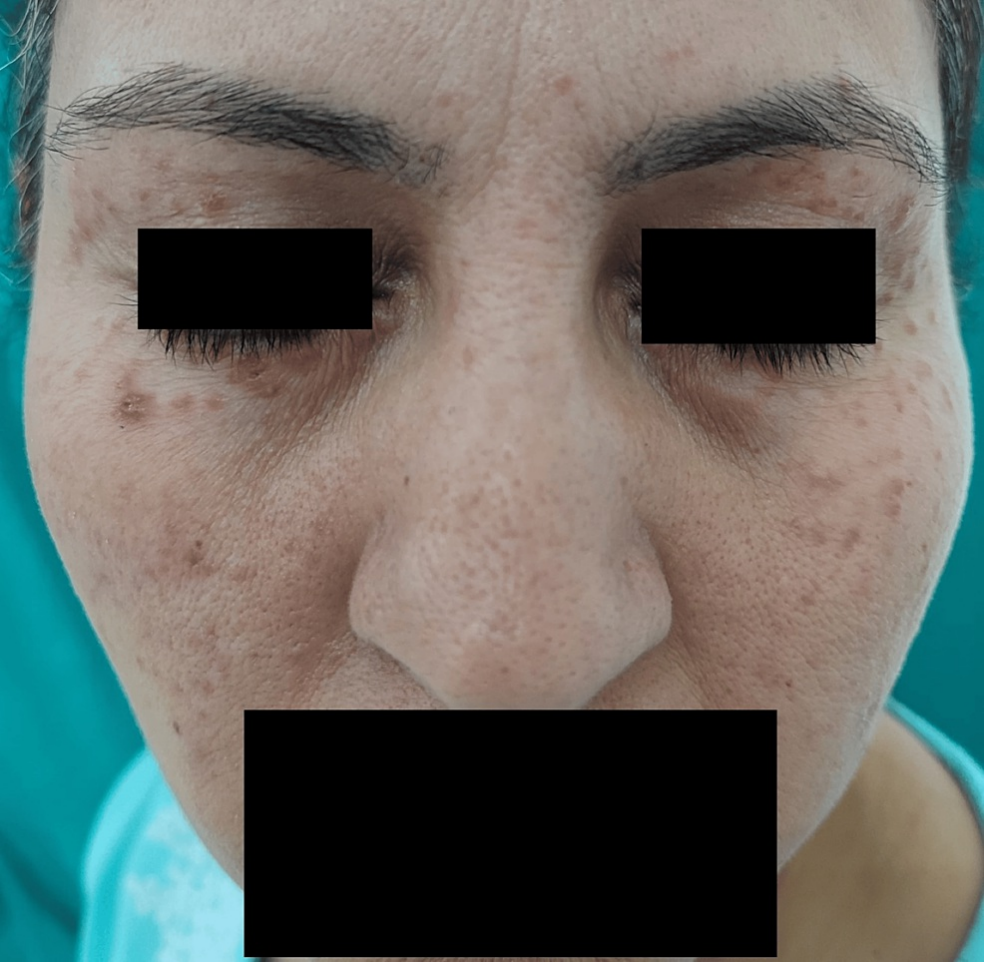
Patient presented with erythematous itchy papules on the face, particularly noticeable in the periorbital region.

A 51-year-old lady presented to the dermatology outpatient clinic with many papules located on the right side of her face, mainly in the malar region. Papules were observed together with pruritic symptoms, which commenced prior to a period of three months. The individual had a few papules displaying varioliform facial scars. The patient reported no previous occurrence of this lesion or any family history of comparable lesions. Upon external inspection, there were red raised bumps visible on the face, particularly around the area surrounding the eyes, with sizes ranging from 2 mm to 4 mm. During the ocular examination, the bulbar conjunctiva, tarsal conjunctiva, and eyeball appeared to be in a normal state (Figure 1).

**Figure 2:**
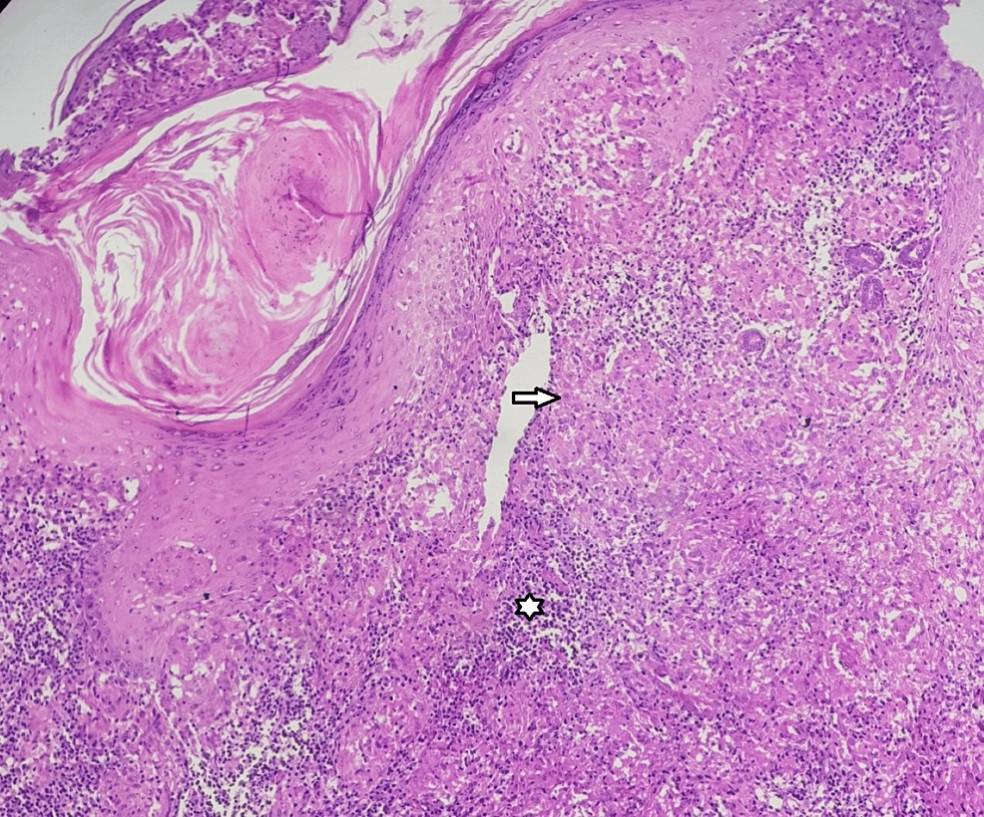
Section shows sub-epidermal collection of inflammatory cells having variably sized granuloma (arrow) with intervening small chronic inflammatory cells (asterisk) (HE stain, 10×).

**Figure 3:**
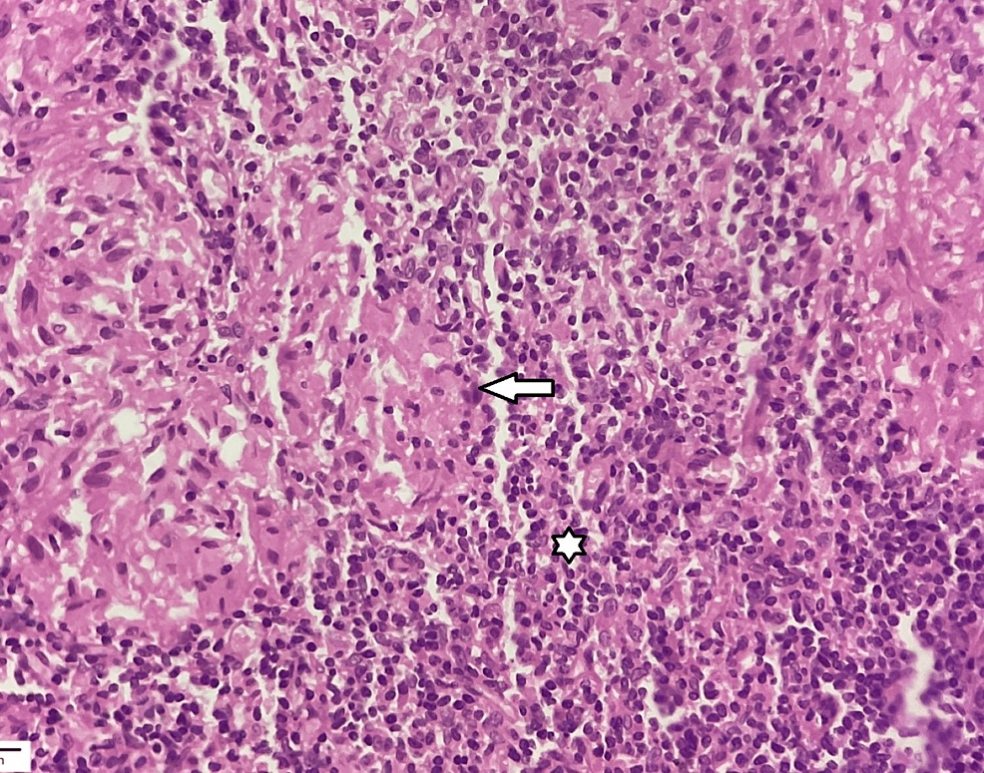
High power view depicting aggregates of epithelioid histiocytes (arrow) surrounded by small lymphocytes (asterisk). Caseating necrosis is not evident in the section (HE stain, 40×).

A punch biopsy was obtained from several papules and forwarded to the pathology laboratory for microscopic examination. The histopathology analysis of the lesion sample revealed dermal proliferation of granulomas, which consisted of epithelioid histiocytes, Langhans giant cells, and lymphocytes. The mononuclear inflammation was observed at both the periadnexal and perivascular sites. The granuloma tested negative for acid-fast bacilli using both Ziehl-Neelsen and Fite-Faraco stains. Additional laboratory tests, such as the complete blood count and biochemistry reports, showed results that were within the normal range (Figures 2-3).

The diagnosis of "LMDF" was made based on histology and the characteristic clinical presentation. The patient was provided with a daily dose of 100 mg of systemic dapsone medication and was also instructed to apply 0.1% topical tacrolimus to the facial lesions twice a day. During the follow-up appointment after four weeks, the lesion exhibited a 50-60% response to therapy, and there was no evidence of any new lesions.

## Discussion

LMDF is an uncommon dermatological condition that mostly affects individuals between the ages of 20 and 40. It is characterized by the presence of numerous tiny, red eruptions or papules over the central area of the face, with a particular tendency to impact the lower eyelid. There have been reports of extra-facial involvement in certain cases. It is alternatively referred to as "acne agminata" or "acnitis." The eruption begins at an early stage and persists in a stable phase for a period of 12-24 months, which may subsequently progress into the formation of disfiguring scars. Nevertheless, it is feasible to reduce the occurrence of scars by initiating prompt therapy [3].

Some writers have suggested that Mycobacterium infection may be a potential etiological cause, as indicated by many synonyms. This hypothesis is not viable due to the absence of any systemic TB, whether in the past or present, as well as the continuous inability to isolate bacilli. An Israeli investigation found that polymerase chain reaction (PCR) was ineffective in detecting mycobacterial DNA in the lesion. As per some reports, the lesion is believed to form due to an abnormal granulomatous reaction to damaged hair follicles [7]. Luo et al. presented a rare instance of Demodex mite-induced LMDF in their case report [8].

The histological hallmark of the LMDF is the presence of epithelioid cell granuloma with central necrosis. Nevertheless, the histological pattern may vary depending on the timing of the biopsy. Initial or emerging lesions may have only the presence of lymphohistiocytic infiltration around blood vessels and the adnexa, without any necrosis. Fully formed classic lesions may exhibit epithelioid cell granulomas, either with or without central necrosis or abscess formation. In histology, late lesions may have perifollicular fibrosis and a nonspecific inflammatory infiltrate. Few cases in the literature report the association of LMDF with ruptured infundibular cysts [6,9,10].

It is essential to take into account various skin disorders characterized by the formation of granulomas. Previously, LMDF was believed to be a form of lupus vulgaris or tuberculid due to its histological similarity. However, there is no evidence to support its connection to TB. Lupus vulgaris, while having a similar histology to LMDF, shows positive results for interferon-gamma release in serum and acid-fast Bacillus (AFB) staining in skin biopsies. As TB progresses, erythematous papules transform into nodules and subsequently develop into ulcers [6].

According to several authors, it is a distinct form of Granulomatous rosacea (GR). This is primarily supported by the evidence that the granulomas in LMDF are connected to pilosebaceous units [6,7] and that certain rosacea individuals have epithelioid granulomas. However, it is challenging to accept this theory due to the apparent differences between the two. LMDF is not classified as a form of rosacea since it is commonly found in young individuals or teens, affects areas beyond the face, particularly the lower eyelids, which are typically unaffected by rosacea, and does not exhibit symptoms such as erythema, flushing, or telangiectasia [1,7,8].

Additionally, LMDF has been proposed as a micropapular variant of sarcoidosis. The pathophysiological features of LMDF, such as the presence of sarcoidal granulomas, as well as the clinical signs and progression, often resemble those observed in cutaneous sarcoidosis. However, the physical examination, chest X-ray, and laboratory testing are usually sufficient to exclude the possibility of sarcoidosis. Sarcoidosis should be evaluated by conducting a blood test to measure elevated levels of angiotensin-converting enzyme, performing a chest X-ray to detect bilateral hilar lymphadenopathy, and conducting a thorough history-taking to assess respiratory and gastrointestinal symptoms. Cutaneous sarcoidosis is characterized by red-brown lesions that have the appearance of apple jelly instead of brilliant erythematous papules [6].

Similarly, perioral dermatitis is characterized by red papules and pustules in the vicinity of the mouth. Given that caseation necrosis is not commonly associated with perioral dermatitis, the diagnosis becomes very straightforward when granulomata are present around the affected region. If biopsy samples only show localized granulomatous inflammation, a thorough clinicopathological correlation may be necessary to establish the diagnosis. The granulomas observed in idiopathic facial aseptic granulomas are classified as foreign body types, and the presence of necrosis is not apparent. This condition, which is usually self-limited and may be associated with granulomatous rosacea, appears in children in the form of facial nodules or papules [6,7].

Other potential differential diagnoses should be considered. Nodulocystic acne is characterized by a variety of lesions, including comedone lesions, and an antibiotic response. An acneiform eruption is associated with a history of drug use before the appearance of lesions. Histoid leprosy is identified by distinct histological features, including spindle-shaped cells and the presence of acid-fast bacilli. Granuloma faciale manifests as a single or a few papules, accompanied by dilated blood vessels and prominent hair follicles on the skin surface. Post-kala-azar dermal leishmaniasis (PKDL) is characterized by papules and nodules on infiltrated skin, associated with hypopigmented lesions. Pseudolymphomas are characterized by lymphoproliferation in histological examination [2,9].

There is currently a scarcity of studies and publications on the therapy of LMDF. Due to the potential remission of the condition within one to two years, reliable evaluation of treatment efficacy is challenging. The available treatment choices include tetracycline, isotretinoin, dapsone, and corticosteroids. Topical medicines, such as tacrolimus (a calcineurin inhibitor) or laser treatment, can be beneficial in treating LMDF. Commencing treatment at an early stage reduces the duration of treatment and minimizes the incidence of scars. Nevertheless, the efficacy of these therapies remains a subject of discussion, and there is presently no standardized therapy protocol [3,5,11]. Tranilast is a recently developed medication for LMDF. Its mechanism of action involves the suppression of collagen production and the inhibition of fibroblast proliferation [9]. Given the superior effectiveness of these therapies compared to others, we administered a combination of systemic dapsone and topical calcineurin inhibitors to the patient in this case.

## Conclusions

LMDF, also known as Lupus miliaris disseminatus faciei, is a rare skin illness of unknown origin. It is defined by certain clinical features and a granulomatous response when examined under a microscope. In order to obtain a definitive diagnosis, it is imperative to exclude other granulomatous illnesses. Timely identification and suitable intervention can help prevent complications such as scarring. Therefore, it is crucial to consider the diagnosis of LMDF when encountering a case with erythematous papules on the central face with a characteristic periorbital position.
